# Low-Cytotoxicity Fluorescent Probes Based on Anthracene Derivatives for Hydrogen Sulfide Detection

**DOI:** 10.3389/fchem.2018.00202

**Published:** 2018-06-05

**Authors:** Xuefang Shang, Jie Li, Yaqian Feng, Hongli Chen, Wei Guo, Jinlian Zhang, Tianyun Wang, Xiufang Xu

**Affiliations:** ^1^Key Laboratory of Medical Molecular Probes, School of Basic Medical Sciences, Xinxiang Medical University, Xinxiang, China; ^2^School of Pharmacy, Xinxiang Medical University, Xinxiang, China; ^3^School of Life Sciences and Technology, Xinxiang Medical University, Xinxiang, China; ^4^Department of Biochemistry, Xinxiang Medical University, Xinxiang, China; ^5^Department of Chemistry, Nankai University, Tianjin, China

**Keywords:** fluorescent probe, hydrogen sulfide, 9-anthracenecarboxaldehyde, nucleophilic substitution, cytotoxicity

## Abstract

Owing to the role of H_2_S in various biochemical processes and diseases, its accurate detection is a major research goal. Three artificial fluorescent probes based on 9-anthracenecarboxaldehyde derivatives were designed and synthesized. Their anion binding capacity was assessed by UV-Vis titration, fluorescence spectroscopy, HRMS, ^1^HNMR titration, and theoretical investigations. Although the anion-binding ability of compound **1** was insignificant, two compounds **2** and **3**, containing benzene rings, were highly sensitive fluorescent probes for HS^−^ among the various anions studied (HS^−^, F^−^, Cl^−^, Br^−^, I^−^, AcO^−^, H_2_${\text{PO}}_{4}^{-}$, ${\text{SO}}_{3}^{2-}$, Cys, GSH, and Hcy). This may be explained by the nucleophilic reaction between HS^−^ and the electron-poor C=C double bond. Due to the presence of a nitro group, compound **3**, with a nitrobenzene ring, showed stronger anion binding ability than that of compound **2**. In addition, compound **1** had a proliferative effect on cells, and compounds **2** and **3** showed low cytotoxicity against MCF-7 cells in the concentration range of 0–150 μg·mL^−1^. Thus, compounds **2** and **3** can be used as biosensors for the detection of H_2_S *in vivo* and may be valuable for future applications.

## Introduction

Hydrogen sulfide (H_2_S) is a toxic gas with smell resembling rotten eggs. It is a bioactive gaseous signaling molecule, along with nitrous oxide (NO) and carbon monoxide (CO) (Kimura et al., 2012; Lisjak et al., 2013; Kimura, 2015; Mishanina et al., 2015). CO and NO are reactive oxygen species, whereas H_2_S gas is a scavenger of reactive oxygen species. Under certain pressure conditions, H_2_S can modulate mitochondria in mammalian cells. It also participates in many biochemical processes such as inflammation, blood pressure control, neuro-transmission, and ischemia reperfusion (Fu et al., 2012; Andreadou et al., 2015; Li F. et al., 2015; Wallace et al., 2015). H_2_S is also a relaxing agent that can act on smooth muscle and can serve as a modulator of cardiac function in cardiovascular therapy (Polhemus and Lefer, 2014; Barr et al., 2015; Chai et al., 2015; Holwerda et al., 2015). In addition, abnormal levels of H_2_S are associated with many diseases, oxygen sensing, and even death (Olson et al., 2006; Pandey et al., 2012). Therefore, the construction of fluorescent probe to detect H_2_S has important practical applications.

Traditional methods for determining the concentration of H_2_S in biological samples include colorimetric, electrochemical, chromatographic, metal-induced vulcanization, and fluorescence analyses (Tangerman, 2009; Shen et al., 2011). Fluorescent molecular probes are commonly used for detection tool in various fields, including in biological samples owing to their ability to convert chemical information into light signals with high sensitivity and selectivity. Hence, the development of fluorescent probes for the detection of H_2_S has attracted substantial research attention (Jiménez et al., 2003; Choi et al., 2009; Yu et al., 2012, 2014).

**Scheme 1:**
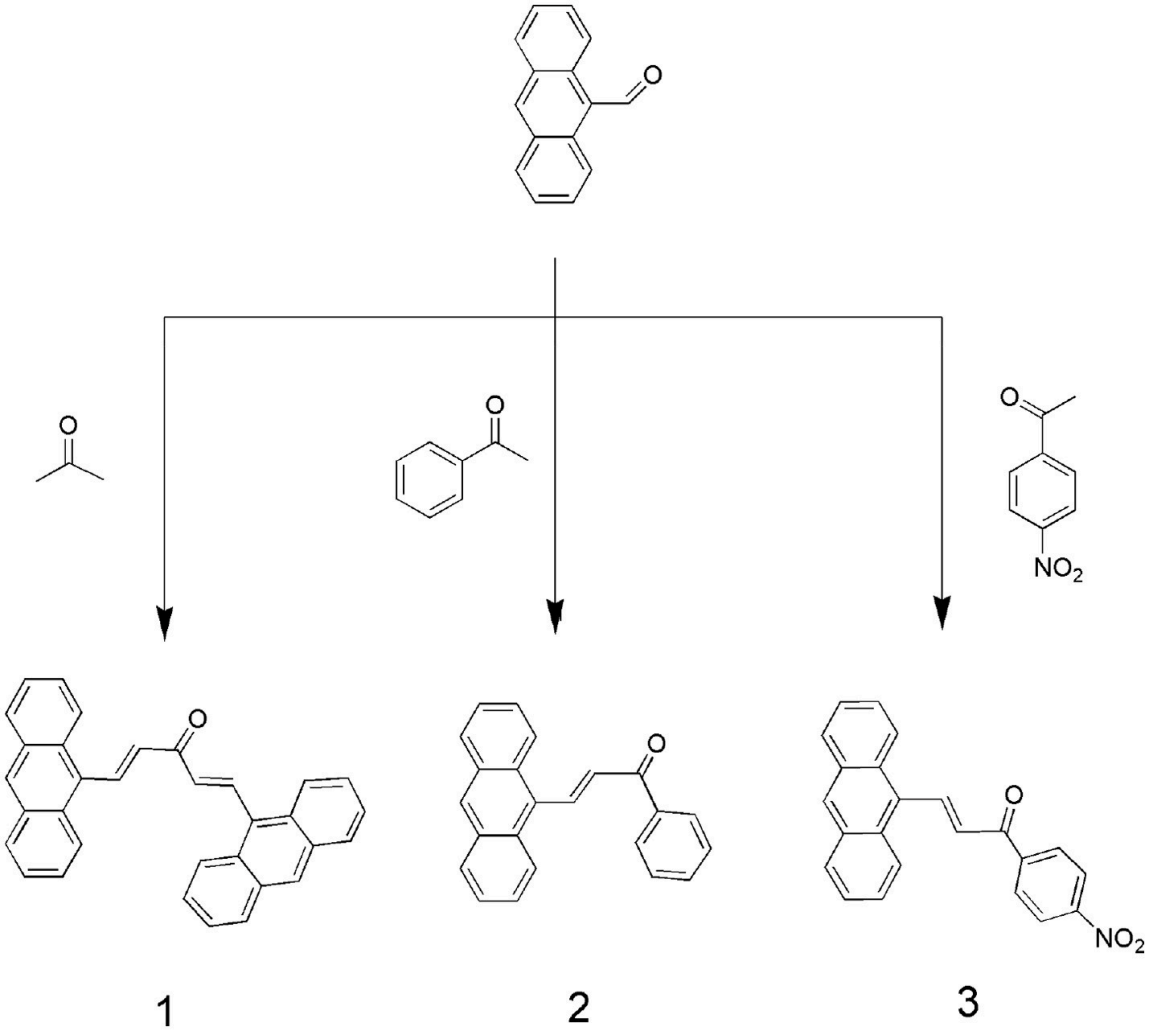
Synthesis routes of compounds **1**, **2**, and **3**.

However, a few reports have focused on the development of fluorescent probes based on the binuclear character of H_2_S (Asthana et al., 2016; Das et al., 2016). Therefore, we used this approach to synthesize highly selective and sensitive fluorescent probes that can detect H_2_S. Under physiological conditions, hydrogen sulfides exist as 30% H_2_S in a non-resolving state and 70% residual HS^−^. Thus, HS^−^ detection can serve as a proxy for H_2_S. In this study, we designed and synthesized novel anthracene derivatives in which a -C = C- bond served as an interaction site (Scheme 1). The abilities of these compounds to bind to various anions (HS^−^, (*n*-C_4_H_9_)_4_NF (F^−^), (*n*-C_4_H_9_)_4_NCl (Cl^−^), (*n*-C_4_H_9_)_4_NBr (Br^−^), (*n*-C_4_H_9_)_4_NI (I^−^), (*n*-C_4_H_9_)_4_NAcO (AcO^−^), (*n*-C_4_H_9_)_4_NH_2_PO_4_ (H_2_${\text{PO}}_{4}^{-}$), Na_2_SO_3_ (${\text{SO}}_{3}^{2-}$), cysteine (Cys), glutathione(GSH), and homocysteine (Hcy) were assessed through UV-Vis titration, fluorescence spectroscopy, HRMS and ^1^HNMR titration for HS^−^ sensitivity and selectivity. These compounds were also investigated for cytotoxicity to MCF-7 cells.

## Materials and methods

Most of the starting materials were obtained commercially. All reagents and solvents were of analytical grade. Sodium hydrosulfide, all anions, in the form of tetrabutylammonium salts such as (*n*-C_4_H_9_)_4_NF, (*n*-C_4_H_9_)_4_NCl, (*n*-C_4_H_9_)_4_NBr, (*n*-C_4_H_9_)_4_NI, (*n*-C_4_H_9_)_4_NAcO, and (*n*-C_4_H_9_)_4_NH_2_PO_4_, and amino acids (Cys, GSH, and Hcy) were purchased from Aladdin (Shanghai, People's Republic of China), stored in a vacuum desiccator containing self-indicating silica, and used without further purification. Tetrabutylammonium salts were dried for 24 h under a vacuum with P_2_O_5_ at 333 K before use. Dimethyl sulfoxide was distilled in vacuo after being dried with CaH_2_. ^1^H NMR spectra were recorded using a Varian Unity Plus 400 MHz spectrometer. ESI-HRMS was performed using a Mariner apparatus. UV-Vis spectroscopy titration was performed using a Shimadzu UV2550 spectrophotometer at 289 K. Fluorometric titration was performed using an Eclipse fluorescence spectrophotometer (Agilent, Santa Clara, CA, USA) at 298 K. IR spectroscopy was performed using an IRTracer-100 instrument. The binding constants (*K*_s_) were obtained by the non-linear least-squares method for data fitting.

Cells in logarithmic growth phase were seeded in 96-well plates at a density of 2.0 × 10^4^ cells per well and cultured for 24 h. The culture medium was then replaced with 200 μL of Roswell Park Memorial Institute (RPMI) 1640 medium containing various concentrations of the compound, and the cells were further incubated for 24 h. Next, the cells were washed with phosphate buffered saline (PBS) three times, and 100 μL of culture medium and 20 μL of MTT solution were added to each well. After further incubation (4 h), the absorbance of each well was detected at 490 nm using a microplate reader (Thermo Multiskan MK3, Thermo Fisher Scientific, MA, USA). Plain cell culture medium was used as the control.

Compound **1** was synthesized according to previous methods (Ding et al., 2013). 9-Anthracenecarboxaldehyde (82.4 mg, 0.4 mmol) and acetone (35 mg, 0.6 mmol) were dissolved in ethanol (50 mL). Then, under stirring, an aqueous sodium hydroxide solution (2 mL, 0.04 mol·L^−1^) was slowly added to the reaction flask. The mixture was stirred at room temperature for 6 h and adjusted to pH 5–6 with dilute hydrochloric acid (0.1 mol·L^−1^) until the reaction was complete. The reaction was monitored by thin-layer chromatography. Typically, a precipitate formed and was collected by filtration. The solid was washed with high purity water and ethanol, and dried under a vacuum. Yield: 87%. ^1^H-NMR (400 MHz, CDCl_3_, 298 K) δ 8.84 (d, J = 16.2 Hz, 1H), 8.52 (s, 1H), 8.38 (d, J = 8.3 Hz, 2H), 8.07 (d, J = 7.9 Hz, 2H), 7.69–7.47 (m, J = 88 Hz, 4H). ^13^C NMR (101 MHz, CDCl_3_) δ 194.10, δ 147.53, δ 141.15, δ 135.40, δ 134.28, δ 129.71, δ 128.98, δ 128.60, δ 126.54, δ125.35. IR spectrum, ν cm ^−1^: 1668 (C = O); 1628 (C = C); 1593 (Ar-C = C); 999 (C = C-H). ESI-HRMS (*m/z*): 457.2 (*M* + Na)^+^.

Compound **2** and **3** were synthesized according to the above procedure.

Compound **2**: ^1^H NMR (400 MHz, CDCl_3_, 298 K) δ 8.83 (d, J = 15.8 Hz, 1H), 8.52 (s, 1H), 8.40–8.27 (m, J = 52 Hz, 2H), 8.18–8.00 (m, J = 72 Hz, 4H), 7.68–7.60 (m, 2H), 7.60–7.48 (m, 6H). ^13^C NMR (101 MHz, DMSO) δ 191.24,δ 140.88,δ 139.87,δ 137.75,δ 131.15,δ 129.15,δ 128.52,δ 127.32,δ 126.53,δ 125.50. IR spectrum, ν cm ^−1^: 3050 (Ar C-H); 1730 (C = O); 1560 (C = C); 720 (C = C-H). ESI-HRMS (*m/z*): 309.1 (*M* + H)^+^, 331.1 (*M* + Na)^+^.

Compound **3**: ^1^H NMR (400 MHz, CDCl_3_, 298 K) δ 8.92 (d, J = 15.8 Hz, 1H), 8.92 (d, J = 15.8 Hz, 1H), 8.55 (s, 1H), 8.47 (s, 1H), 8.51–8.36 (m, J = 60.0 Hz, 3H), 8.42–8.22 (m, 6H), 8.28 (dd, J = 23.9 Hz, 8.3 Hz, 4H), 8.16–8.05 (m, J = 44 Hz, 2H), 8.15–8.04 (m, J = 44 Hz, 2H), 7.62–7.52 (m, J = 40 Hz, 4H), 7.65–7.52 (m, J = 52 Hz, 4H), 7.28 (s, 3H). ^13^C NMR (101 MHz, DMSO) δ 188.64,δ 150.36,δ 142.55,δ 131.59,δ 131.32,δ 129.47,δ 127.43,δ 126.15,δ 125.55,δ 124.41. IR spectrum, ν cm^−1^: 1750 (C = O); 1590 (C = C); 1520 (N-O); 880 (C = N). ESI-HRMS (*m/z*): 376.1 (*M* + Na)^+^.

## Results and discussion

### UV-Vis spectral titration

**Figure 1:**
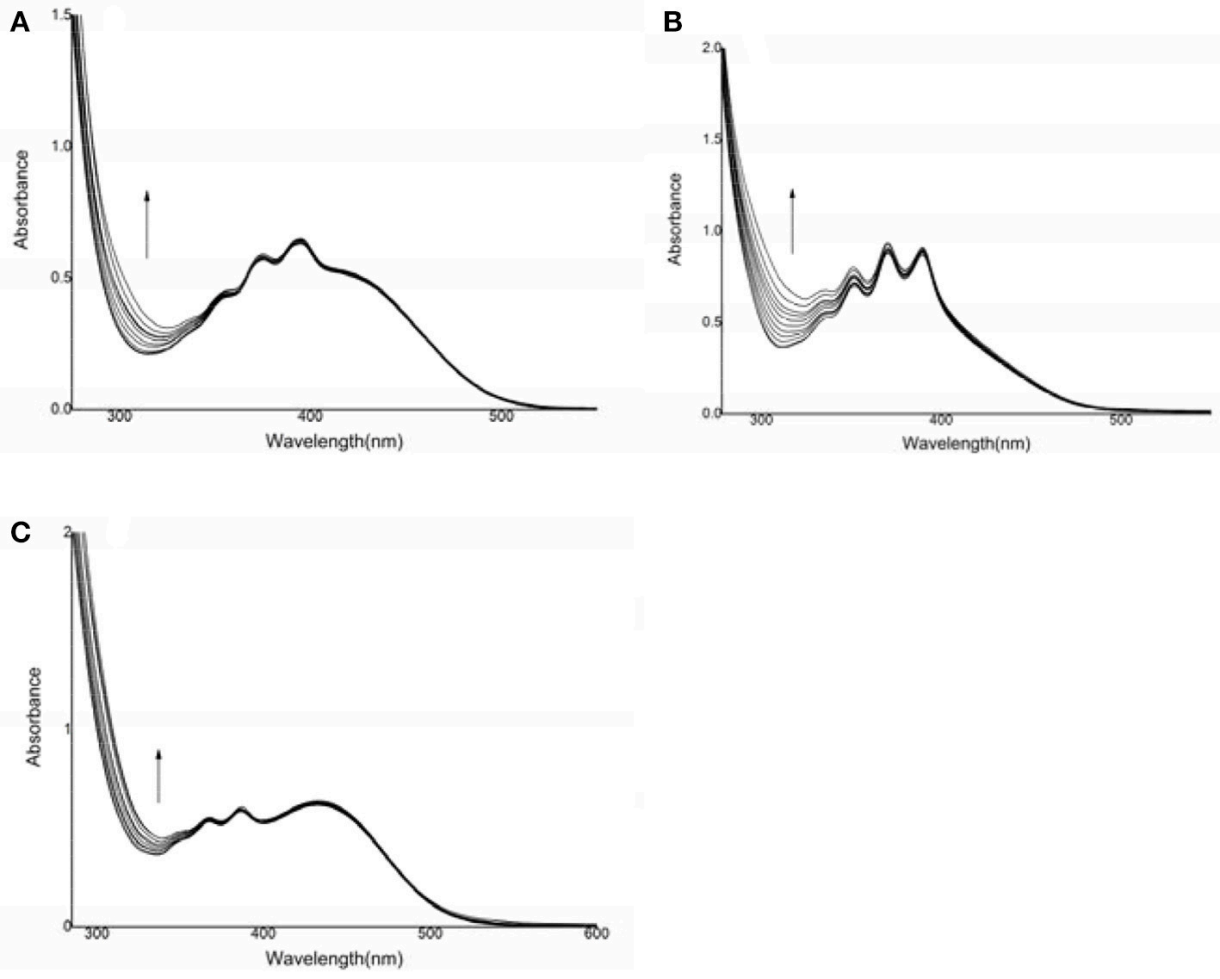
UV-vis spectral changes of compounds **1**, **2**, and **3** after the addition of HS^−^. **(A)** compound **1**: 6.90 × 10^−5^ mol·L^−1^, HS^−^: (0–76) × 10^−5^ mol·L^−1^; **(B)** compound **2**: 1.46 × 10^−4^ mol·L^−1^, HS^−^: (0–2) × 10^−3^ mol·L^−1^; **(C)** compound **3**: 1.1 × 10^−4^ mol·L^−1^, HS^−^: (0–16) × 10^−4^ mol·L^−1^.

UV-Vis titration was performed in dimethyl sulfoxide by the stepwise addition of sodium hydrosulfide (Figure 1). For compound **1**, the presence of HS^−^ resulted in an increase in the absorption intensity at 315 nm, but the spectral changes were very small. Furthermore, the addition of F^−^, Cl^−^, Br^−^, I^−^, AcO^−^, H_2_${\text{PO}}_{4}^{-}$, ${\text{SO}}_{3}^{2-}$, Cys, GSH, or Hcy resulted in very weak spectral changes for compound **1**, and the binding capacity was negligible.

For compound **2**, the intensity of the absorption peak increased at 312 nm after the addition of sodium hydrosulfide. A hyperchromic effect was observed during the host-guest interaction process. The change in the UV-Vis spectrum was due to the interaction between sodium hydrosulfide and the electron-deficient C = C double bond (Zhao et al., 2012). However, the addition of F^−^, Cl^−^, Br^−^, I^−^, AcO^−^, or H_2_${\text{PO}}_{4}^{-}$ did not cause a substantial spectral response for compound **2** (Figure S1), suggesting that the host-guest interaction was weak (Shao et al., 2009; Shang et al., 2013, 2015a). For compound **3**, the intensity of the absorption peak at 336 nm increased, and the absorption band was enhanced after HS^−^ addition. However, the addition of F^−^, Cl^−^, Br^−^, I^−^, AcO^−^, H_2_${\text{PO}}_{4}^{-}$, ${\text{SO}}_{3}^{2-}$, Cys, GSH, or Hcy resulted in a very weak spectral response, indicating that the host-guest interaction was negligible. These results suggested that compounds **2** and **3** both showed high sensitivity and selectivity for HS^−^.

### Fluorescence response

**Figure 2:**
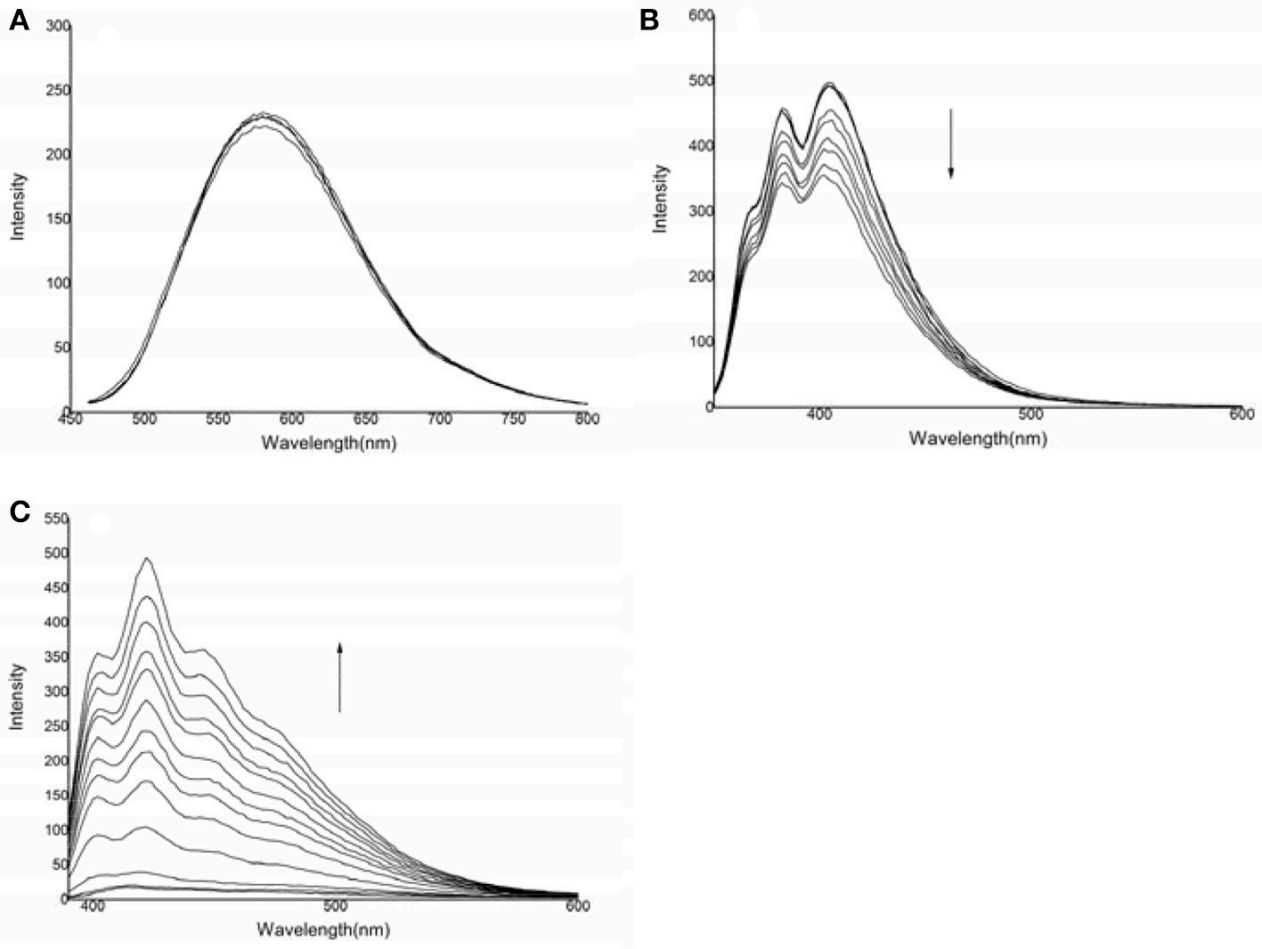
Changes in the emission spectra of the three compounds in the presence of HS^−^: **(A)** compound **1**: 6.9 × 10^−5^ mol·L^−1^, HS^−^: 0–20.7 × 10^−5^ mol·L^−1^, λ_ex_ = 442 nm; **(B)** compound **2**: 1.46 × 10^−4^ mol·L^−1^, HS^−^: 0–50.1 × 10^−4^ mol·L^−1^, λ_ex_ = 324 nm; **(C)** compound **3**: 1.1 × 10^−4^ mol·L^−1^, HS^−^: 0–7.7 × 10^−4^ mol·L^−1^, λ_ex_ = 368 nm.

The photophysical responses of the three probes to various anions were examined. As shown in Figure 2, compound **1** showed an emission peak centered at 582 nm. After the addition of HS^−^ to a solution of compound **1**, the spectral response of compound **1** was very weak, indicating that the binding ability was negligible.

For compound **2**, emission peaks were centered at 382 and 404 nm. After the addition of HS^−^, the fluorescence emission was significantly quenched. No significant spectral changes were observed after titration of F^−^, Cl^−^, Br^−^, I^−^, H_2_${\text{PO}}_{4}^{-}$, AcO^−^, ${\text{SO}}_{3}^{2-}$, Cys, GSH, or Hcy, indicating that compound **2** had an insignificant binding capacity for these anions (Figure S2A).

For compound **3**, there was almost no fluorescence response. After the addition of HS^−^, a new emission peak at approximately 420 nm appeared, which was gradually accompanied by two shoulders centered at 402 and 440 nm. This fluorescence enhancement may be resulted from two possible signal transduction mechanisms: the inhibition of photo-electron transfer and binding induced by the guest's host molecules (Watanabe et al., 1998; Lee et al., 2002; Lin et al., 2006). However, no significant spectral changes were observed when compound **3** was titrated with F^−^, Cl^−^, Br^−^, I^−^, H_2_${\text{PO}}_{4}^{-}$, AcO^−^, ${\text{SO}}_{3}^{2-}$, Cys, GSH, or Hcy, indicating that compound **3** did not significantly bind to these anions (Figure S2B). The fluorescence calibration curve for compound **3** after the addition of HS^−^ indicated that the emission intensity was non-linear when various quantities of HS^−^ were added to a solution with a certain concentration of compound **3** (Shang et al., 2012a).

### Binding constant

The spectral responses of compound **1** after the addition of anions were very weak; hence, the binding constant could not be calculated. The UV-Vis spectral changes for compounds **2** and **3** were ascribed to the formation of host-guest (1:2) complexes; when the absorbance intensity was greatest, the ratio of [H]/([H]+[G]) was approximately 0.3, according to a Job-plot (Figure S3). The binding constants were calculated by the non-linear least-squares method according to the UV-Vis data provided in Table 1 (Bourson et al., 1993; Liu et al., 2001, 2004). It was shown that, the spectra changed little for compound **1**, and compounds **2** and **3** showed the strongest binding ability for HS^−^ among the various anions tested. The anion binding abilities were in decreasing order: HS^−^ >> ${\text{SO}}_{3}^{2-}$ ~ Cys ~ GSH ~ Hcy ~ F^−^ ~ Cl^−^ ~ Br^−^ ~ I^−^ ~ AcO^−^ ~ H_2_${\text{PO}}_{4}^{-}$. The standard deviations for the binding constants were *R*_3_ = 0.9941 and *R*_2_ = 0.9945. Among the three compounds, the standard deviation for compound **1** was not statistically significant, and those for compounds **2** and **3** were significant (compound **2**, **S** = 31.6011, compound **3**, **S** = 159.3298) (Figure S6). The anion binding ability could be attributed to the host-guest interactions and the match in space structures. It means that HS^−^ ions strongly bound to these compounds, according to their binding constants (Shang et al., 2012b).

**Table 1:** Binding constants of the three compounds with various anions.

**Anion^a^**	**K_s_ (1)**	**K_s_ (2)**	**K_s_ (3)**
HS^−^	ND^b^	(4.77 ± 0.77) × 10^5^	(1.07 ± 0.45) × 10^6^
F^−^, Cl^−^, Br^−^, I^−^, AcO^−^, H_2_${\text{PO}}_{4}^{-}$, ${\text{SO}}_{3}^{2-}$, Cys, GSH, Hcy	ND	ND	ND

a *Anions was added in the form of sodium sulfide or tetra-n-butylammonium salts*.

b *The spectra changed little, and the binding constant could not be determined (ND)*.

**Scheme 2:**
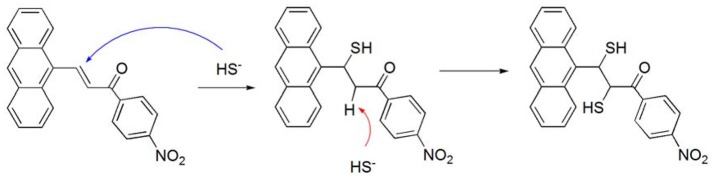
The possible interaction mechanism between compound **3** and HS^−^.

Compound **3** showed a stronger binding ability toward HS^−^ ions than that of compound **2**, owing to the presence of a nitro group. The nitro group served as an electron-withdrawing group that enhanced the binding ability between the C = C double bond in compound **3** and HS^−^. According to the HRMS data, the observed negative ion peak (418.0577) was the MS peak of the **3**-HS^−^ complex (theoretical value: 418.0572) (Figure S4). In addition, there was no peak of –CH_2_- in the ^1^HNMR titration results, suggesting that the C = C double bond was broken during the interaction between compound **3** and HS^−^ (Figure S5). Therefore, a possible host-guest binding mechanism was as follows. The first step was the Michael addition reaction of the conjugated system (Li J. et al., 2015). The first HS^−^ ion was added to the C = C moiety as a nucleophile. Then, the second HS^−^ ion attacked the active hydrogen atom (alpha-H) as an electrophile moiety, forming the final structure as shown in Scheme 2. The final structure was verified by mass spectrometry. The reaction of compound **3** with HS^−^ was conducted in a simulated physiological environment, and the reaction product was subjected to a fluorescence analysis. A large increase in the fluorescence spectrum was observed.

### Cytotoxicity assessment

**Figure 3:**
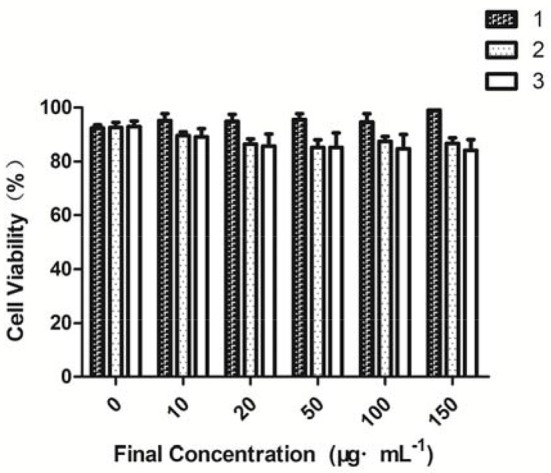
Cell viability values (%), as estimated from MTT proliferation assays, vs. incubation concentrations of fluorescent probe.

The cytotoxicity of the three compounds against MCF-7 cells was evaluated by MTT assays (Vibet et al., 2008; Jiang et al., 2014; Alemany et al., 2015; Jouvin et al., 2015; Moustakim et al., 2017) (Figure 3). Compound **1** had a proliferative effect on the cells, and compounds **2** and **3** in the range of 0–150 μg·mL^−1^ showed very low cytotoxicity. Cell viability was minimally affected (80% cell viability), when the concentrations of compounds **2** and **3** were increased to 150 μg·mL^−1^. In agreement with the determined binding constants, compounds **2** and **3** each showed a high binding capacity and low cytotoxicity and thus can be used to detect HS^−^ *in vivo* (Gao et al., 2015; Shang et al., 2017). Compared with previous estimates in the literature (Zou et al., 2013; Lin et al., 2015), the cytotoxicity of the synthesized compounds was relatively low. Hence, these probes are favorable candidates for *in vitro* hydrogen sulfide detection.

### Theoretical investigation

**Figure 4:**
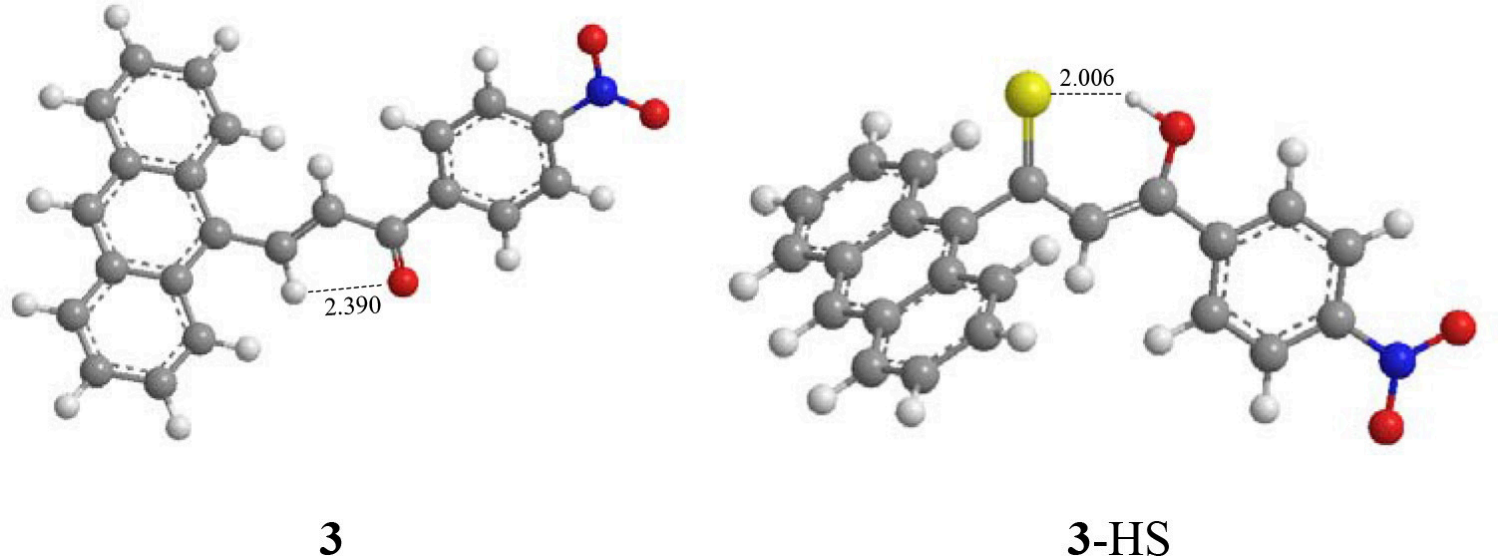
Optimized geometries of compound **3** and the combination product **3**-HS.

Among the three synthesized compounds, compound **3** showed the highest sensitivity and selectivity for HS^−^ according to the binding constants. Consequently, the geometries were optimized for compound **3** and the combination product **3**-HS (Figure 4) based on the density functional theory method and the level of B3LYP/3-21G. The calculation was implemented in Gaussian03 (Frisch et al., 2003; Gao et al., 2017). As shown in Figure 4, the distance of the intramolecular hydrogen bond in compound **3** was 2.390 Å between the hydrogen atom of the interaction site (-HC = CH-) and the oxygen atom of the carbonyl group. According to previous studies (Ni et al., 2012; Maity et al., 2014), the existence of intramolecular hydrogen bonding and an electron-withdrawing group (-NO_2_) increases the sensitivity. Hence, the stronger the electron-withdrawing effect is, the higher sensitivity for HS^−^ this compound gets. The combination between compound **3** and HS^−^ was also optimized. Our results indicated that the spatial structure of the host may change, as a result of the host-guest interaction. Therefore, the combination product (**3**-HS) existed in resonance form. The distance of the hydrogen bond (2.006 Å) indicated that a stable six-cycle was formed containing a sulfur atom and a hydrogen atom in a hydroxyl group (the resonance form of ketone) after compound **3** interacted with HS^−^. These results also explained the strong ability of compound **3** to bind to HS^−^.

**Figure 5:**
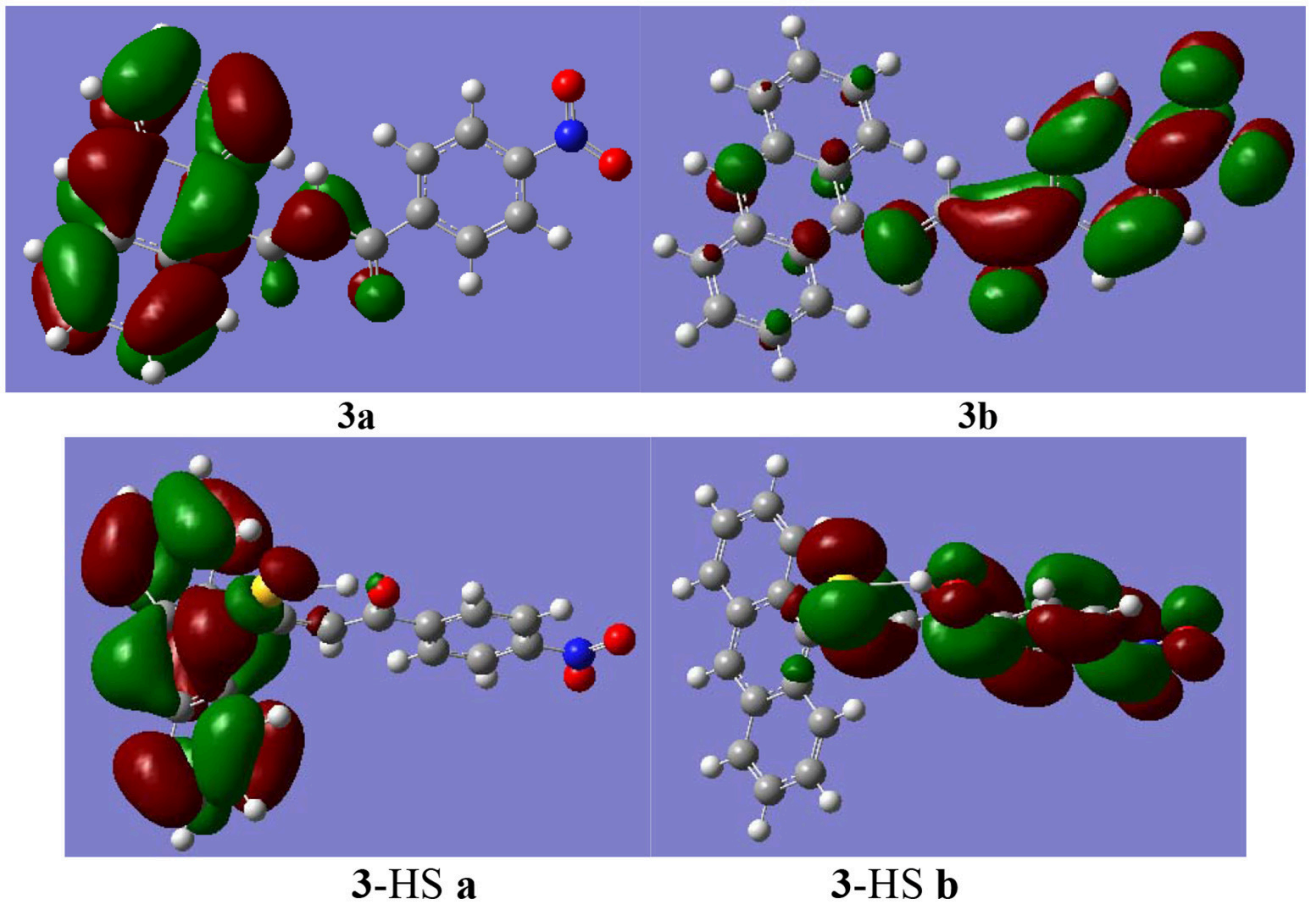
The selected molecular frontier orbitals HOMO **(a)** and LUMO **(b)**.

In addition, the molecular frontier orbitals were introduced to explore the hyperchromic effect (by UV-Vis titration as described above). This effect was observed in the host-guest interaction process by the electron transition of the frontier orbital. The selected frontier orbitals for compound **3** and the host-guest complex are shown in Figure 5. An orbital analysis revealed that the highest occupied molecular orbital (HOMO) density in compound **3** was mainly localized on the anthracene moiety, whereas the lowest unoccupied molecular orbital (LUMO) density was localized on the nitrophenyl and ketone group moieties (Shang et al., 2015b). These results indicated that the electron transition of the highest HOMO resulted in a hyperchromic effect in the UV-Vis spectra.

## Conclusions

In conclusion, three compounds were synthesized, and their abilities to bind to various anions were detected by UV-Vis titration, fluorescence spectroscopy, HRMS, ^1^HNMR titration and theoretical investigations. Compounds **2** and **3** showed selectivity and sensitivity for HS^−^. Notably, compound **3** showed the strongest sensing ability for HS^−^ among the synthesized compounds. The mechanism underlying this interaction was the nucleophilic reaction between HS^−^ and the electron-poor C = C double bond. Theoretical investigations also elucidated the role of molecular frontier orbitals in the hyperchromic effect. In addition, compounds **2** and **3** showed low cytotoxicity against MCF-7 cells in the concentration range of 0–150 μg·mL^−1^ and can be subsequently used as fluorescent probes to detect H_2_S, HS^−^, or S^2−^ species *in vivo*. These results provide a probe with a novel sensing mechanism for hydrogen sulfide, based on the amphipolar character of the S atom of the new compounds to be used in practical applications to detect H_2_S. Our finding establishes a basis for further applications of molecular probes.

## Author contributions

XS, and TW responsible for the experimental design. JL and YF responsible for the synthesis and properties of detection. WG and JZ responsible for the characterization of compounds. HC is responsible for the detection of cytotoxicity. XX is responsible for the quantitative calculation of the data.

### Conflict of interest statement

The authors declare that the research was conducted in the absence of any commercial or financial relationships that could be construed as a potential conflict of interest.

## References

[B1] Alemany L. Saunier M. Alvarado-Cabrero I. Quirós B. Salmeron J. Shin H. R.. (2015). Human papillomavirus DNA prevalence and type distribution in anal carcinomas worldwide. Int. J. Cancer 136, 98–107. 10.1002/ijc.28963 24817381 PMC4270372

[B2] Andreadou I. Iliodromitis E. K. Szabo C. Papapetropoulos A. (2015). Hydrogen sulfide and PKG in ischemia–reperfusion injury: sources, signaling, accelerators and brakes. Basic Res. Cardiol. 110:52. 10.1007/s00395-015-0510-9 26318600 PMC4667708

[B3] Asthana S. K. Kumar A. Upadhyay K. K. (2016). Efficient visualization of H_2_S via a fluorescent probe with three electrophilic centres. Org. Biomol. Chem. 14, 3690–3694. 10.1039/C6OB00197A 27030288

[B4] Barr L. A. Shimizu Y. Lambert J. P. Nicholson C. K. Calvert J. W. (2015). Hydrogen sulfide attenuates high fat diet-induced cardiac dysfunction via the suppression of endoplasmic reticulum stress. Nitric Oxide 46, 145–156. 10.1016/j.niox.2014.12.03 25575644 PMC4361249

[B5] Bourson J. Pouget J. Valeur B. (1993). Ion-responsive fluorescent compounds. 4. Effect of cation binding on the photophysical properties of a coumarin linked to monoaza-and diaza-crown ethers. J. Phys. Chem. 97, 4552–4557. 10.1021/j100119a050

[B6] Chai Q. Lu T. Wang X. L. Lee H. C. (2015). Hydrogen sulfide impairs shear stress-induced vasodilation in mouse coronary arteries. Pflügers Arch. Eur. J. Physiol. 467, 329–340. 10.1007/s00424-014-1526-y 24793048

[B7] Choi M. G. Cha S. Lee H. Jeon H. L. Chang S. K. (2009). Sulfide-selective chemosignaling by a Cu^2+^ complex of dipicolylamine appended fluorescein. Chem. Commun. 7390–7392. 10.1039/B916476F 20024238

[B8] Das A. K. Goswami S. Dutta G. Maity S. Kanti Mandal T. Khanra K.. (2016). A concentration dependent auto-relay-recognition by the same analyte: a dual fluorescence switch-on by hydrogen sulfide via Michael addition followed by reduction and staining for bio-activity. Org. Biomol. Chem. 14, 570–576. 10.1039/C5OB02008E 26510406

[B9] Ding J. Ge Y. Zhu B. (2013). A highly selective fluorescent probe for quantitative detection of hydrogen sulfide. Anal. Sci. 29, 1171–1175. 10.2116/analsci.29.1171 24334983

[B10] Frisch M. J. Trucks G. W. Schlegel H. B. Scuseria G. E. Robb M. A. Cheeseman J. R. (2003). Gaussian 03, revision A. 1. Pittsburgh, PA: Gaussian Inc.

[B11] Fu M. Zhang W. Wu L. Yang G. Li H. Wang R. (2012). Hydrogen sulfide (H_2_S) metabolism in mitochondria and its regulatory role in energy production. Proc. Natl. Acad. Sci. U.S.A. 109, 2943–2948. 10.1073/pnas.1115634109 22323590 PMC3287003

[B12] Gao M. Yu F. Chen H. Chen L. (2015). Near-infrared fluorescent probe for imaging mitochondrial hydrogen polysulfides in living cells and *in vivo*. Anal. Chem. 87, 3631–3638. 10.1021/ac5044237 25751615

[B13] Gao M. Yu F. Lv C. Choo J. Chen L. (2017). Fluorescent chemical probes for accurate tumor diagnosis and targeting therapy. Chem. Soc. Rev. 46, 2237–2271. 10.1039/C6CS00908E 28319221

[B14] Holwerda K. M. Karumanchi S. A. Lely A. T. (2015). Hydrogen sulfide: role in vascular physiology and pathology. Curr. Opin. Nephrol. Hypertens. 24, 170–176. 10.1097/MNH.0000000000000096 25587904

[B15] Jiang P. Liu Q. Liang Y. Tian J. Asiri A. M. Sun X. (2014). A cost-effective 3D hydrogen evolution cathode with high catalytic activity: FeP nanowire array as the active phase. Angew. Chem. Int. Ed Engl. 53, 12855–12859. 10.1002/anie.201406848 25257101

[B16] Jiménez D. Martínez-Máñez R. Sancenón F. Ros-Lis J. V. Benito A. Soto J. (2003). A new chromo-chemodosimeter selective for sulfide anion. J. Am. Chem. Soc. 125, 9000–9001. 10.1021/ja0347336 15369341

[B17] Jouvin K. Matheis C. Goossen L. J. (2015). Synthesis of aryl tri- and difluoromethyl thioethers via a C-H-thiocyanation/fluoroalkylation cascade. Chemistry 21, 14324–14327. 10.1002/chem.201502914 26332514

[B18] Kimura H. (2015). Signaling molecules: hydrogen sulfide and polysulfide. Antioxid. Redox Signal. 22, 362–376. 10.1089/ars.2014.5869 24800864 PMC4307032

[B19] Kimura H. Shibuya N. Kimura Y. (2012). Hydrogen sulfide is a signaling molecule and a cytoprotectant. Antioxid. Redox Signal. 17, 45–57. 10.1089/ars.2011.4345 22229673 PMC3342561

[B20] Lisjak M. Teklic T. Wilson I. D. Whiteman M. Hancock J. T. (2013). Hydrogen sulfide: environmental factor or signalling molecule? Plant Cell Environ. 36, 1607–1616. 10.1111/pce.12073 23347018

[B21] Lee D. H. Im J. H. Lee J. H. Hong J. I. (2002). A new fluorescent fluoride chemosensor based on conformational restriction of a biaryl fluorophore. Tetrahedron Lett. 43, 9637–9640. 10.1016/S0040-4039(02)02443-7

[B22] Li F. Zhang P. Zhang M. Liang L. Sun X. Bao A. (2015). Effects of hydrogen sulfide on ozone-induced features of chronic obstructive pulmonary disease. Eur. Respir. J. 46(suppl59):PA4114 10.1183/13993003

[B23] Li J. Yin C. Huo F. (2015). Chromogenic and fluorogenic chemosensors for hydrogen sulfide: review of detection mechanisms since the year 2009. RSC Adv. 5, 2191–2206. 10.1039/C4RA11870G

[B24] Lin V. S. Chen W. Xian M. Chang C. J. (2015). Chemical probes for molecular imaging and detection of hydrogen sulfide and reactive sulfur species in biological systems. Chem. Soc. Rev. 44, 4596–4618. 10.1039/C4CS00298A 25474627 PMC4456340

[B25] Lin Z. H. Zhao Y. G. Duan C. Y. Zhang B. G. Bai Z. P. (2006). A highly selective chromo-and fluorogenic dual responding fluoride sensor: naked-eye detection of F^−^ ion in natural water via a test paper. Dalton Trans. 3678–3684. 10.1039/B601282E 16865180

[B26] Liu Y. Han B. H. Zhang H. Y. (2004). Spectroscopic studies on molecular recognition of modified cyclodextrins. Curr. Org. Chem. 8, 35–46. 10.2174/1385272043486061

[B27] Liu Y. You C. C. Zhang H. Y. (2001). Supramolecular Chemistry. Tian Jin, Nankai University Publication.

[B28] Maity D. Bhaumik C. Mondal D. Baitalik S. (2014). Photoinduced intramolecular energy transfer and anion sensing studies of isomeric Ru II Os II complexes derived from an asymmetric phenanthroline–terpyridine bridge. Dalton Trans. 43, 1829–1845. 10.1039/C3DT52186A 24257479

[B29] Mishanina T. V. Libiad M. Banerjee R. (2015). Biogenesis of reactive sulfur species for signaling by hydrogen sulfide oxidation pathways. Nat. Chem. Biol. 11, 457–464. 10.1038/nchembio.1834 26083070 PMC4818113

[B30] Moustakim M. Clark P. G. Trulli L. Fuentes de Arriba A. L. Ehebauer M. T. Chaikuad A.. (2017). Discovery of a PCAF bromodomain chemical probe. Angew. Chem. Int. Ed Engl. 56, 827–831. 10.1002/anie.201610816 27966810 PMC5412877

[B31] Ni X. L. Tahara J. Rahman S. Zeng X. Hughes D. L. Redshaw C.. (2012). Ditopic Receptors based on lower-and upper-rim substituted hexahomotrioxacalix [3] arenes: cation-controlled hydrogen bonding of anion. Chem. Asian J. 7, 519–527. 10.1002/asia.201100926 22246650

[B32] Olson K. R. Dombkowski R. A. Russell M. J. Doellman M. M. Head S. K. Whitfield N. L.. (2006). Hydrogen sulfide as an oxygen sensor/transducer in vertebrate hypoxic vasoconstriction and hypoxic vasodilation. J. Exp. Biol. 209, 4011–4023. 10.1242/jeb.02480 17023595

[B33] Pandey S. K. Kim K. H. Tang K. T. (2012). A review of sensor-based methods for monitoring hydrogen sulfide. TrAC Trends Anal. Chem. 32, 87–99. 10.1016/j.trac.2011.08.008

[B34] Polhemus D. J. Lefer D. J. (2014). Emergence of hydrogen sulfide as an endogenous gaseous signaling molecule in cardiovascular disease. Circ. Res. 114, 730–737. 10.1161/CIRCRESAHA 24526678 PMC3951140

[B35] Shang X. Hao Y. Wang Y. Han J. Zhai Y. Jia S. (2012a). Influence of different substituents on anion binding ability in aromatic hydroxyl group derivatives: experiment and theory. Curr. Anal. Chem. 8, 392–399. 10.2174/157341112801264950

[B36] Shang X. Li J. Guo K. Ti T. Wang T. Zhang J. (2017). Development and cytotoxicity of Schiff base derivative as a fluorescence probe for the detection of l-Arginine. J. Mol. Struct. 1134, 369–373. 10.1016/j.molstruc.2016.12.105

[B37] Shang X. Li W. Wei X. Zhang H. Fu Z. Zhang J. (2015a). Synthesis, Bioactivity, and the Anion-Binding Property of 2-Sulfydryl-1, 3, 4-thiodiazole Derivatives. Heteroatom Chem. 26, 142–149. 10.1002/hc.21239

[B38] Shang X. Li X. Li C. Wang Y. Zhang J. Xu X. (2012b). Spectral response to oxy-anions based on ferrocenylphalene. Inorgan. Chim. Acta 385, 128–134. 10.1016/j.ica.2012.01.044

[B39] Shang X. Luo L. Ren K. Wei X. Feng Y. Li X.. (2015b). Synthesis and cytotoxicity of azo nano-materials as new biosensors for l-Arginine determination. Mater. Sci. Eng. C 51, 279–286. 10.1016/j.msec.2015.03.005 25842136

[B40] Shang X. Tian S. Xi N. Li Y. Liu Y. Yin Z.. (2013). Colorimeric and fluorescence ON–OFF probe for acetate anion based on thiourea derivative: theory and experiment. Spectrochim. Acta Part A Mol. Biomol. Spectrosc. 103, 276–281. 10.1016/j.saa.2012.11.027 23261623

[B41] Shao J. Yu X. Xu X. Lin H. Cai Z. Lin H. (2009). Colorimetric and fluorescent sensing of biologically important fluoride in physiological pH condition based on a positive homotropic allosteric system. Talanta 79, 547–551. 10.1016/j.talanta.2009.02.023 19559919

[B42] Shen X. Pattillo C. B. Pardue S. Bir S. C. Wang R. Kevil C. G. (2011). Measurement of plasma hydrogen sulfide *in vivo* and *in vitro*. Free Radic. Biol. Med. 50, 1021–1031. 10.1016/j.freeradbiomed.2011.01.025 21276849 PMC4798232

[B43] Tangerman A. (2009). Measurement and biological significance of the volatile sulfur compounds hydrogen sulfide, methanethiol and dimethyl sulfide in various biological matrices. J. Chromatogr. B 877, 3366–3377. 10.1016/j.jchromb.2009.05.026 19505855

[B44] Vibet S. Goupille C. Bougnoux P. Steghens J. P. Goré J. Mahéo K. (2008). Sensitization by docosahexaenoic acid (DHA) of breast cancer cells to anthracyclines through loss of glutathione peroxidase (GPx1) response. Free Radic. Biol. Med. 44, 1483–1491. 10.1016/j.freeradbiomed.2008.01.009 18267129

[B45] Wallace J. L. Blackler R. W. Chan M. V. Da Silva G. J. Elsheikh W. Flannigan K. L.. (2015). Anti-inflammatory and cytoprotective actions of hydrogen sulfide: translation to therapeutics. Antioxid. Redox Signal. 22, 398–410. 10.1089/ars.2014.5901 24635322

[B46] Watanabe S. Onogawa O. Komatsu Y. Yoshida K. (1998). Luminescent metalloreceptor with a neutral bis (acylaminoimidazoline) binding site: optical sensing of anionic and neural phosphodiesters. J. Am. Chem. Soc. 120, 229–230. 10.1021/ja973263a

[B47] Yu F. Han X. Chen L. (2014). Fluorescent probes for hydrogen sulfide detection and bioimaging. Chem. Commun. 50, 12234–12249. 10.1039/C4CC03312D 25029966

[B48] Yu F. Li P. Song P. Wang B. Zhao J. Han K. (2012). An ICT-based strategy to a colorimetric and ratiometric fluorescence probe for hydrogen sulfide in living cells. Chem. Commun. 48, 2852–2854. 10.1039/C2CC17658K 22293939

[B49] Zhao Y. Zhu X. Kan H. Wang W. Zhu B. Du B. Zhang X. (2012). A highly selective colorimetric chemodosimeter for fast and quantitative detection of hydrogen sulfide. Analyst 137, 5576-5580. 10.1039/C2AN36106J 23057070

[B50] Zou Q. Fang Y. Zhao Y. Zhao H. Wang Y. Gu Y. Wu F. (2013). Synthesis and *in vitro* photocytotoxicity of coumarin derivatives for one-and two-photon excited photodynamic therapy. J. Med. Chem. 56, 5288-5294. 10.1021/jm400025g 23763331

