# Supplementary material for: Low-Cytotoxicity Fluorescent Probes Based on Anthracene Derivatives for Hydrogen Sulfide Detection
Source: Front Chem. 2018 Jun 5;6:202. doi: 10.3389/fchem.2018.00202 (PMC6024568; doi:10.3389/fchem.2018.00202)
Supplement: Supplementary file 1 [file Data_Sheet_1.doc]

Supplementary Information

**Low-cytotoxicity fluorescent probes based on anthracene derivatives for hydrogen sulfide detection**

Xuefang Shang^a^*, Jie Li^a^, Yaqian Feng^b^, Hongli Chen^c^, Wei Guo^a^, Jinlian Zhang^b^, Tianyun Wang^d^, Xiufang Xu^e^

^a^Department of Chemistry, Xinxiang Medical University, Xinxiang, Henan 453003, China

^b^School of Pharmacy, Xinxiang Medical University, Xinxiang, Henan 453003 China

^c^School of Life Sciences and Technology, Xinxiang Medical University, Jinsui Road 601, Xinxiang, Henan 453003 China

^d^Department of biochemistry, Xinxiang Medical University, Jinsui Road 601, Xinxiang, Henan 453003, China

^e^Department of Chemistry, Nankai University, Tianjin 300071, China

*Corresponding author: Tel +86-373-3029128, Fax +86-373-3029959

E-mail: xuefangshang@126.com

**Spectroscopic Data**


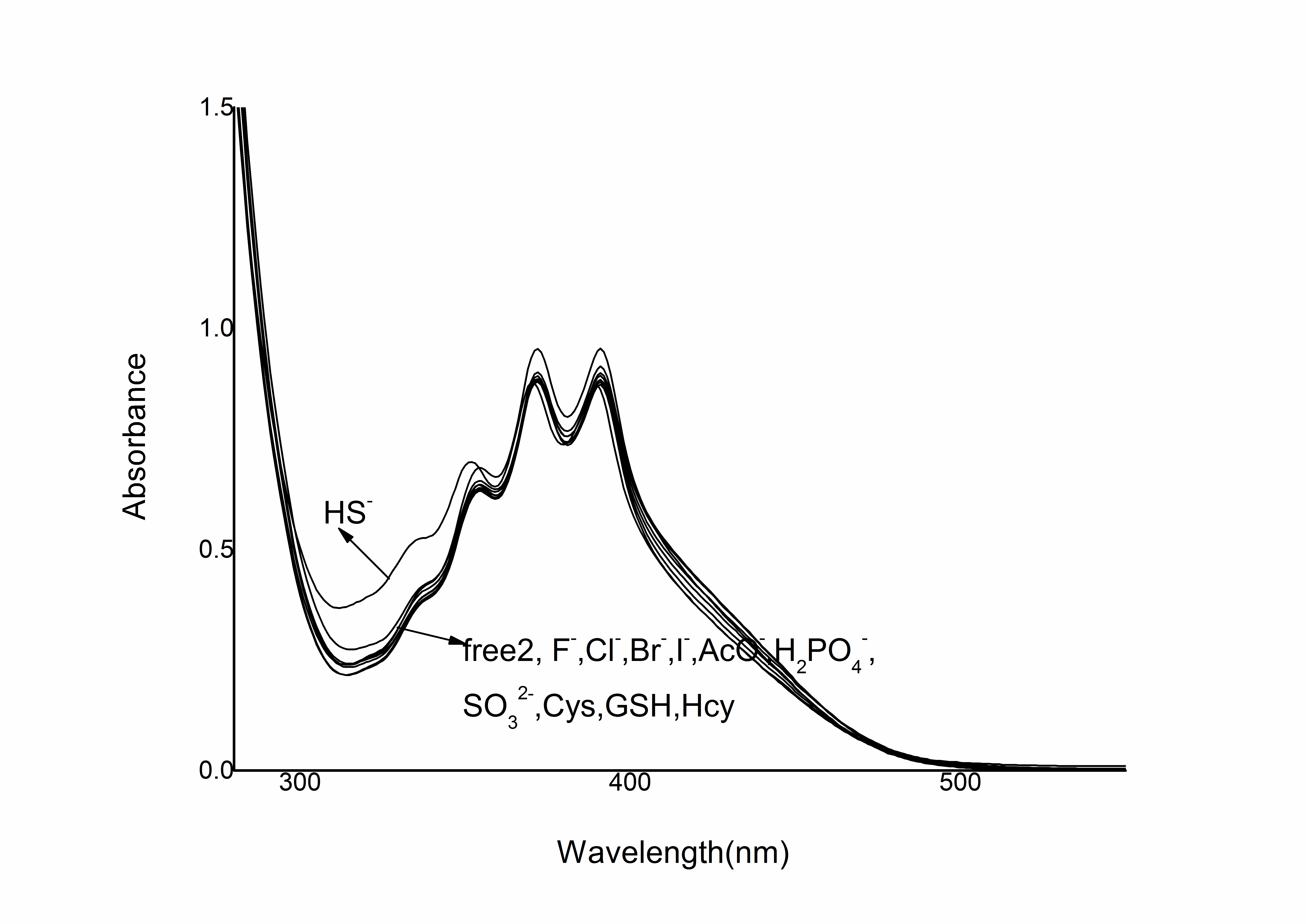


**Figure S1** UV-vis spectral changes of compound **2** (1.46 × 10^-4^ mol·L^-1^) upon the addition of anions (2.0 × 10^-3^ mol·L^-1^).

**
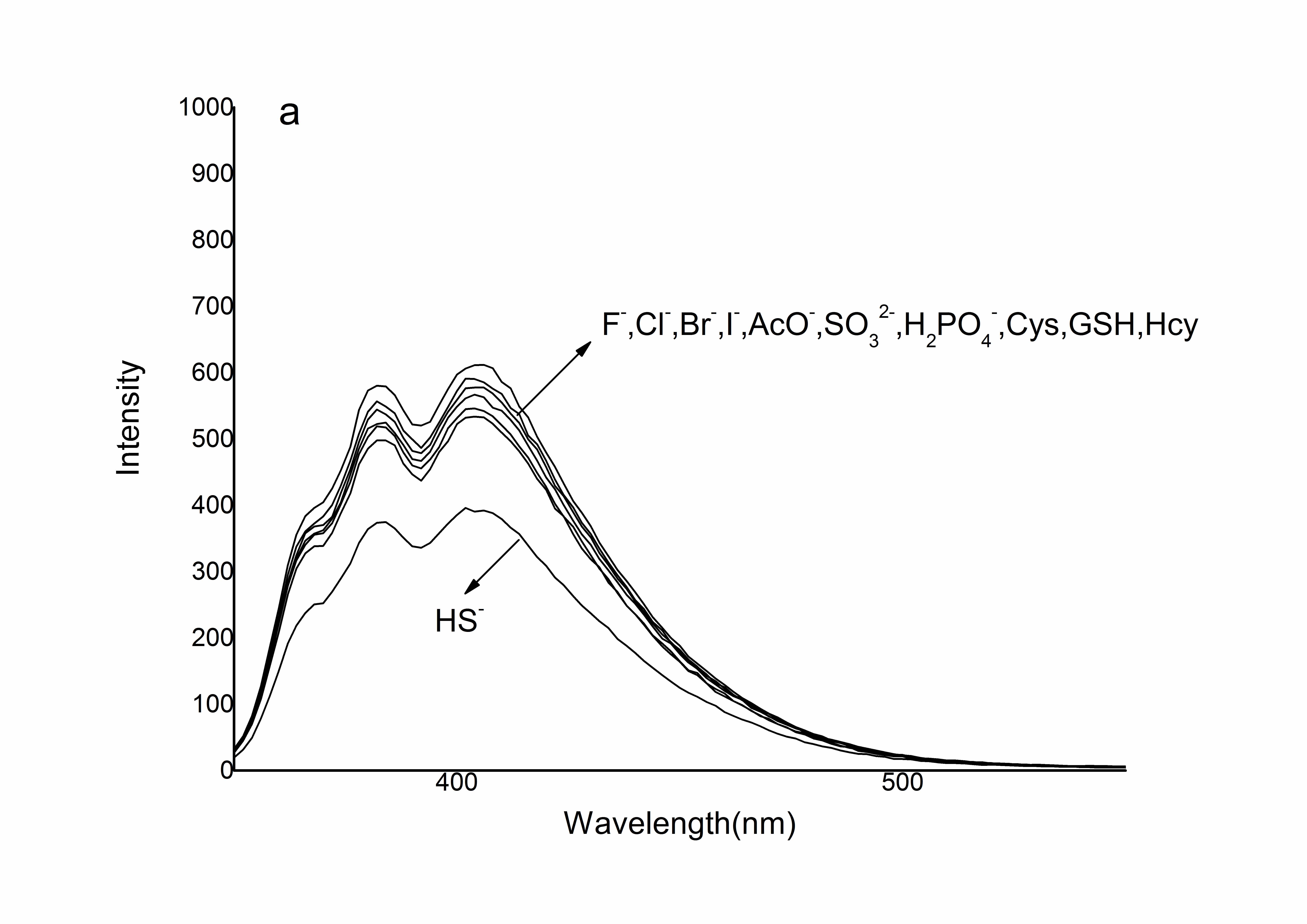
**

**Figure S2** a) Fluorescence changes of compound **2** (1.46 × 10^-4^ mol·L^-1^) upon the addition of anions (2.19×10^-3^ mol·L^-1^).

**
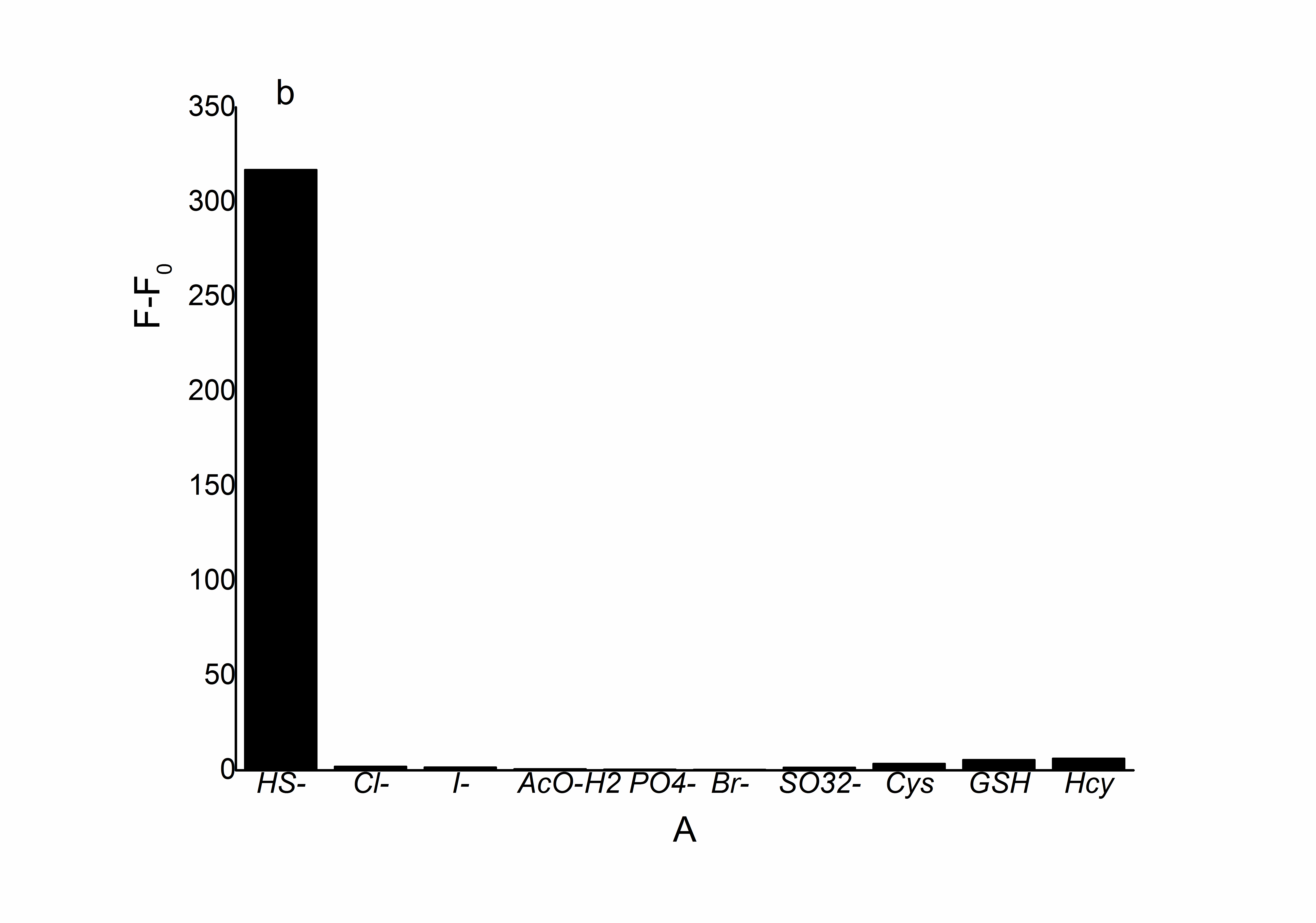
**

**Figure S2** b) Fluorescence changes of compound **3** (1.10 × 10^-4^ mol·L^-1^) upon the addition of anions (5.50 × 10^-4^ mol·L^-1^).


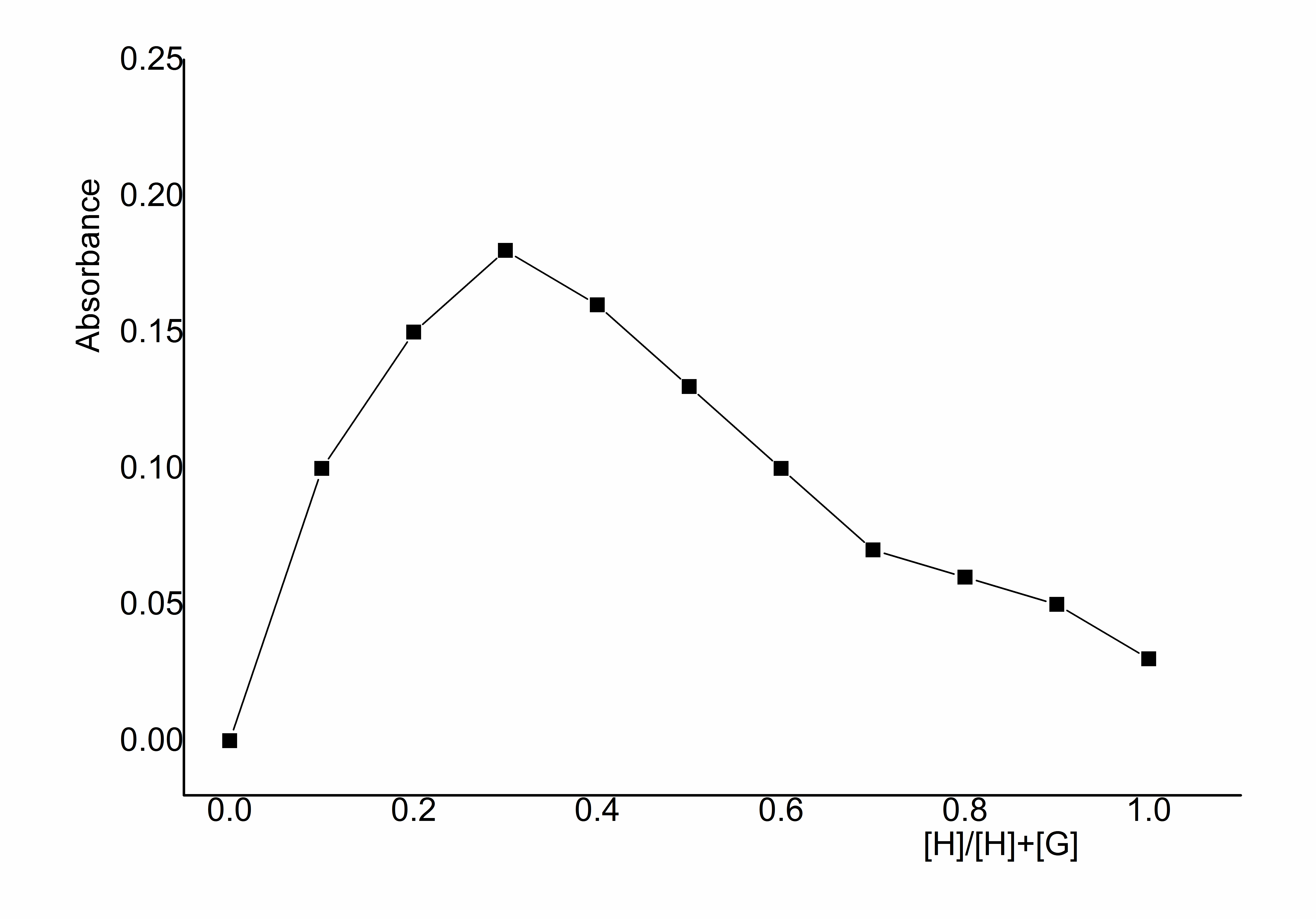


**Figure S3** Job-plot curve of compound **2** upon the addition of HS^−^.

a


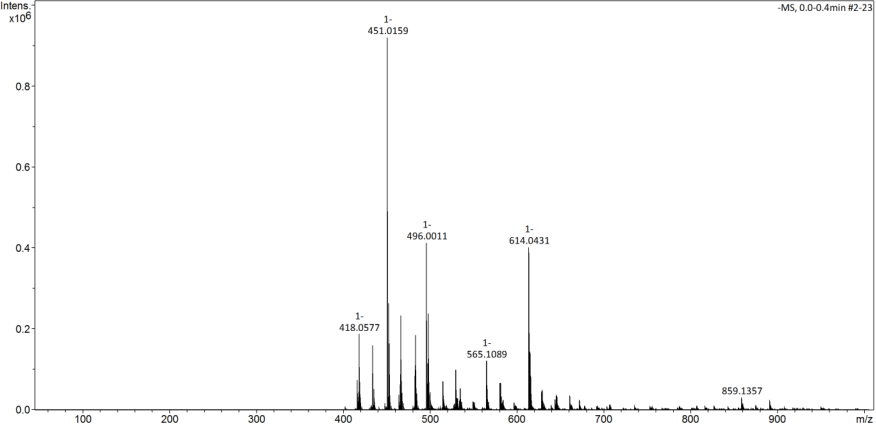


b


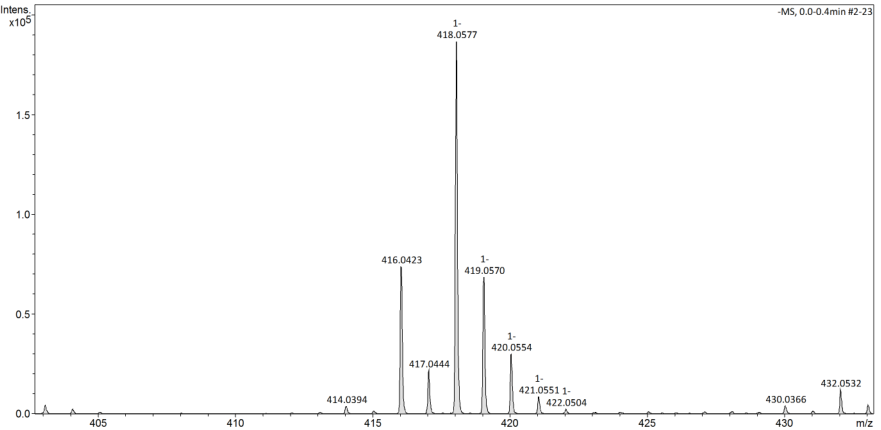


**Figure S4** HRMS of the combination complex (**3**-HS^−^), the concentration ratio of probe **3** to HS^-^ is 1:2; **a** was the full spectrum of **3**-HS^-^; **b** was the magnification of **3**-HS^-^


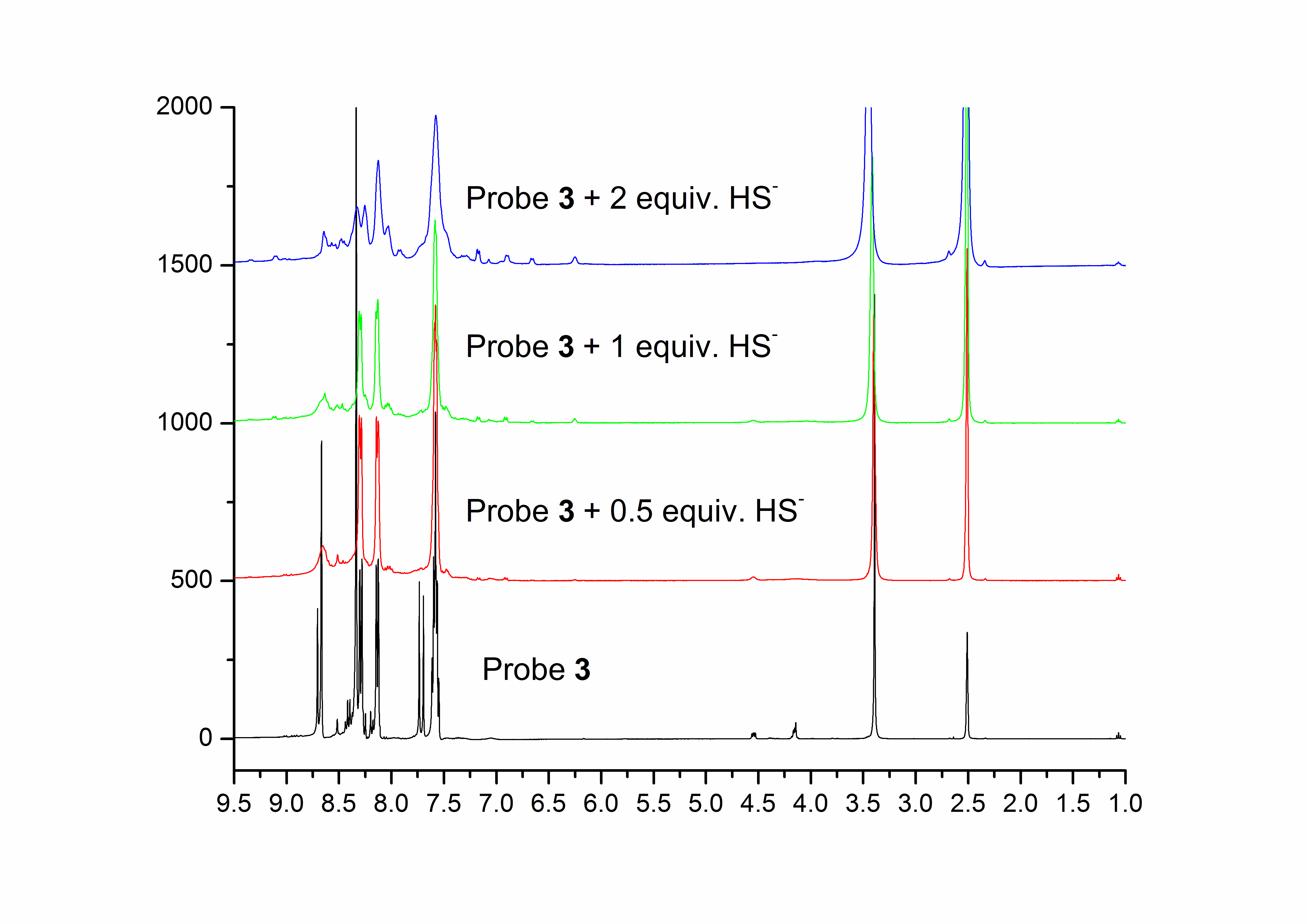


**Figure S5**. Partial ^1^HNMR titration in *d*_6_-DMSO of probe **3** upon the addition of HS^−^.

a


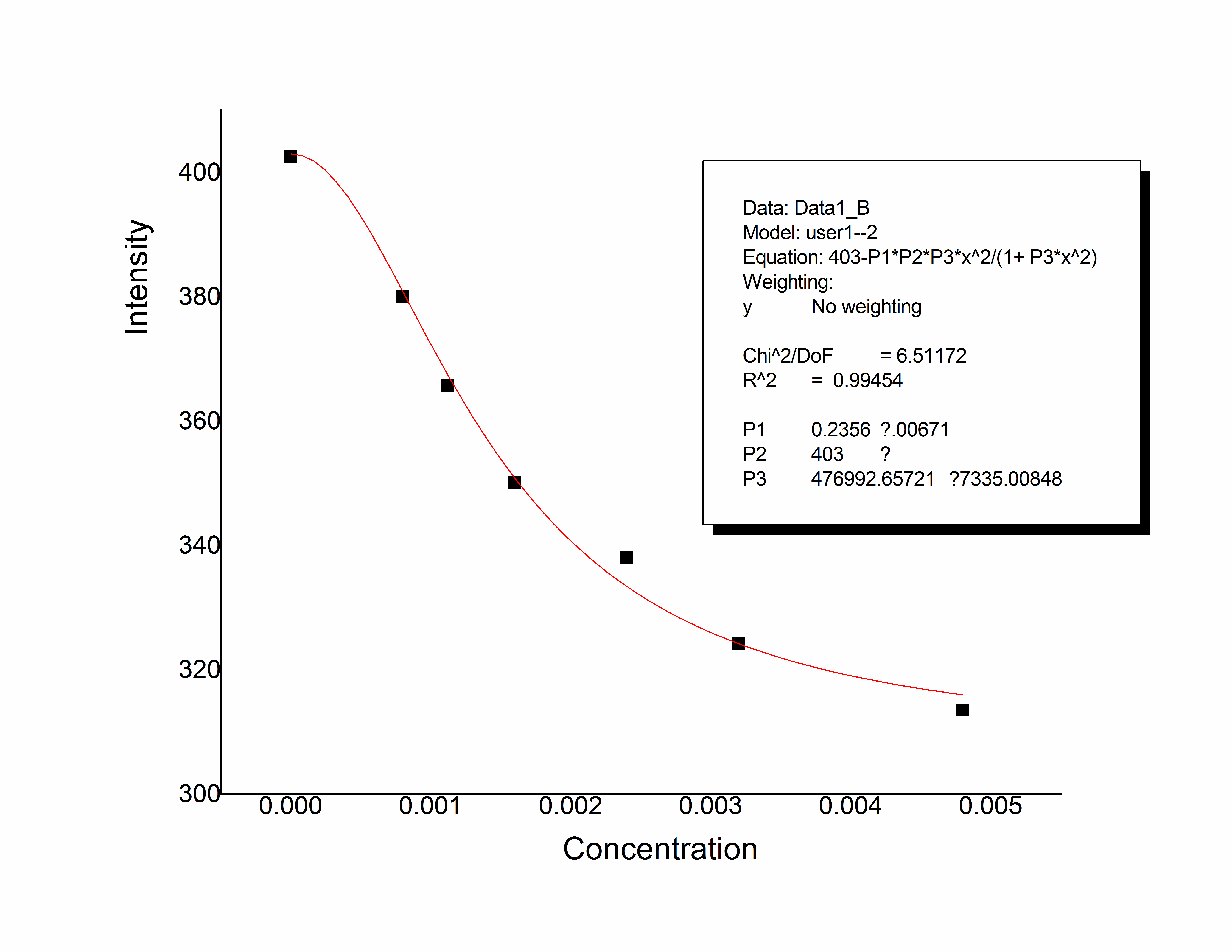


b


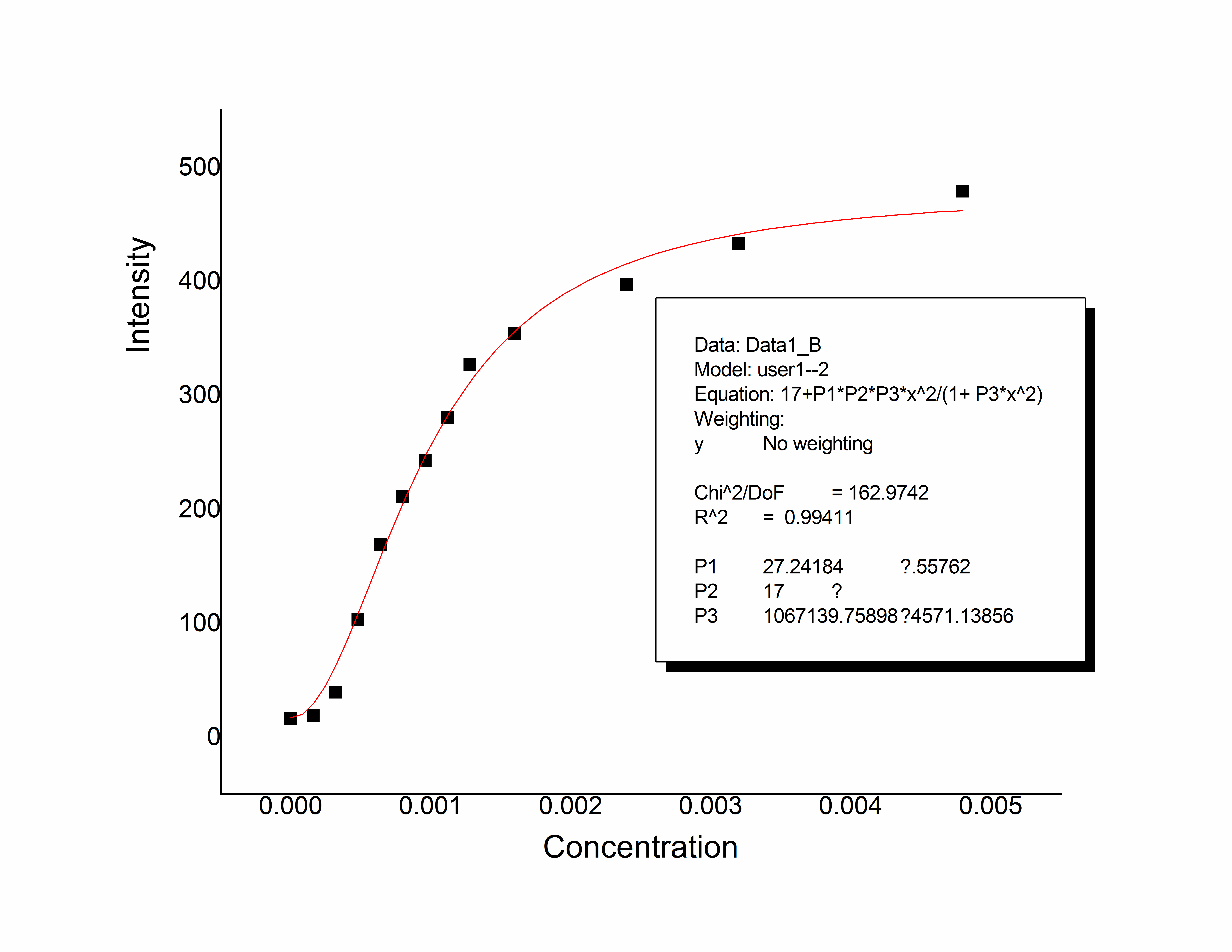


**Figure S6**. a) Non-linear fitting constant plot for compound **2**; b) Non-linear fitting constant plot for compound **3**.
